# UROPOT: study protocol for a randomized, double-blind phase I/II trial for metabolism-based potentiation of antimicrobial prophylaxis in the urological tract

**DOI:** 10.1186/s13063-024-08526-7

**Published:** 2024-10-15

**Authors:** Kevin Stritt, Beat Roth, Audrey Masnada, Felix Hammann, Damien Jacot, Sonia Domingos-Pereira, François Crettenand, Perrine Bohner, Isabelle Sommer, Emilien Bréat, Julien Sauser, Laurent Derré, Manuel Haschke, James J. Collins, John McKinney, Sylvain Meylan

**Affiliations:** 1 Department of Urology, Lausanne University Hospital, Lausanne, Switzerland; 2 Department of Urology, University Hospital of Bern, Inselspital, University of Bern, Bern, Switzerland; 3 Division of Clinical Pharmacology & Toxicology, Department of Internal Medicine, University Hospital Bern, Bern, Switzerland; 4 Institute of Microbiology, Lausanne University Hospital and University of Lausanne, Lausanne, Switzerland; 5 Urology Research Unit and Urology Biobank, Department of Urology, Lausanne University Hospital, Lausanne, Switzerland; 6 Service of Pharmacy, Lausanne University Hospital and University of Lausanne, Lausanne, Switzerland; 7 Centre for Research and Innovation in Clinical Pharmaceutical Sciences, Lausanne University Hospital and University of Lausanne, Lausanne, Switzerland; 8 Clinical Trial Unit, Centre Hospitalier Universitaire Vaudois, Lausanne, Switzerland; 9 Infectious Disease and Microbiome Program, Broad Institute of MIT and Harvard, Cambridge, MA USA; 10 Institute for Medical Engineering and Scienceand, Department of Biological Engineering , Massachusetts Institute of Technology, Cambridge, MA USA; 11 Wyss Institute for Biologically Inspired Engineering, Harvard University, Boston, MA USA; 12 School of Life Sciences, Swiss Federal Institute of Technology in Lausanne (EPFL), Lausanne, 1015 Switzerland; 13 Infectious Diseases Service, Internal Medicine, Lausanne University Hospital and University of Lausanne, Lausanne, Switzerland

**Keywords:** Antimicrobial prophylaxis, Endourological procedures, Postoperative infections, Biofilm, Potentiated aminoglycosides

## Abstract

**Background:**

Urinary tract catheters, including Double-J or ureteral stents, are prone to bacterial colonization forming biofilms and leading to asymptomatic bacteriuria. In the context of asymptomatic bacteriuria, endourological procedures causing mucosa-inducing lesions can lead to severe infections. Antibiotic prophylaxis is warranted, yet its efficacy is limited by biofilm formation on stents. Biofilms promote antibiotic tolerance, the capacity of genetically susceptible bacteria to survive a normally lethal dose of antimicrobial therapy. The UROPOT study evaluates the effectiveness of a first-in-type metabolism-based aminoglycoside potentiation for (i) preventing infectious complications of asymptomatic bacteriuria during mucosa lesion-inducing endourological procedures and (ii) assessing its anti-tolerance efficacy.

**Methods:**

The UROPOT trial is a phase I/II single-center (Lausanne University Hospital (CHUV), Switzerland) randomized double-blinded trial. Over 2 years, patients with asymptomatic *Escherichia coli* and/or *Klebsiella pneumoniae* bacteriuria, undergoing endourological procedures, will be randomly allocated to one of three treatment arms (1:1:1 randomization ratio, 30 patients per group) to evaluate the efficacy of mannitol-potentiated low-dose amikacin compared to established standard treatments (ceftriaxone or amikacin standard dose). Patients will be recruited at the CHUV Urology Outpatient Clinic. The primary outcome is the comparative incidence of postoperative urinary tract infections (assessed at 48 h) between the investigational amikacin/mannitol therapy and standard (ceftriaxone or amikacin) antibiotic prophylaxis, defined by specific systemic symptoms and/or positive blood and/or urine culture. Secondary outcomes include assessing microbiological eradication through anti-biofilm activity, sustained microbiological eradication, and mannitol and antibiotics pharmacokinetics in blood and urine. Safety outcomes will evaluate the incidence of adverse events following amikacin/mannitol therapy and postoperative surgical complications at postoperative day 14.

**Discussion:**

UROPOT tests a novel antimicrobial strategy based on “metabolic potentiation” for prophylaxis enabling aminoglycoside dose reduction and targeting biofilm activity. The anti-biofilm effect may prove beneficial, particularly in patients who have a permanent stent in situ needing recurrent endourological manipulations strategies in preventing infections and achieving sustained microbiological eradication in pre-stented patients.

**Trial registration:**

The protocol is approved by the local ethics committee (CER-VD, 2023–01369, protocole 2.0) and the Swiss Agency for Therapeutic Products (Swissmedic, 701,676) and is registered on the NIH’s ClinicalTrials.gov (trial registration number: NCT05761405). Registered on March 07, 2023.

**Supplementary Information:**

The online version contains supplementary material available at 10.1186/s13063-024-08526-7.

## Introduction

### Background and rationale

Over the past two decades, minimally invasive endourological techniques have become the primary approach for managing urinary stones and obstructive pathologies, often requiring stent placement for extended periods. However, these stents are prone to colonization by urogenital flora, leading to biofilm formation and asymptomatic bacteriuria, with *Escherichia coli* and *Klebsiella pneumoniae* representing over 75% of pathogens [1]. Although asymptomatic bacteriuria itself is not harmful per se, it can escalate to severe infections like urosepsis during mucosal lesion-inducing endourological procedures (e.g., stone fragmentation and/or extraction, stent manipulations) [2, 3]. During endourological interventions, bacteria from ureteral stents or in suspension may spread systemically due to increased intraluminal pressure, potentially leading to symptomatic urinary tract infections (UTI) [4]. In patients with long-lasting or permanent stents, such as cancer patients with upper urinary tract obstruction, over 30% experienced at least one episode of urosepsis during stent exchanges [5–8]. Management of these infectious complications is challenging, exacerbated by the rise of extended spectrum beta-lactamase (ESBL) and multi-drug resistant bacterial strains, complicating antibiotic treatment and prophylaxis [9].

Due to the high risk of infectious complications, the Infectious Disease Society of America (IDSA) recommends a urine culture prior to manipulation to guide antibiotic prophylaxis, with third-generation cephalosporins or carbapenems being frequently used [7, 10]. However, use of beta-lactams is fraught with the development of resistance, necessitating novel strategies to alleviate pressure on first-line agents and decrease the use of broad-spectrum carbapenems [11]. This has prompted the use of aminoglycosides as standard antibiotic therapy for UTIs in Australia (*sahealth.sa.gov.au*). However, none of these regimens is effective against biofilms formed on foreign bodies such as stents [10, 12].

Biofilm-associated bacteria exhibit “antibiotic tolerance,” and thus survive otherwise lethal doses of antibiotics, highlighting the need for effective strategies. While quinolones have an anti-biofilm activity, resistance in Enterobacteriaceae like *E. coli* or *K. pneumoniae* approaches 20% in Switzerland (*anresis.ch*), limiting their utility in urinary tract infection management. Addressing the lack of biofilm eradication strategies, rising antibiotic resistance, and the steady increase in endourological interventions following previous ureteral stenting, the UROPOT study aims to evaluate the clinical significance of a novel antimicrobial strategy during mucosal lesion-inducing procedures in pre-stented patients with asymptomatic bacteriuria.

Previous research in a murine catheter-associated urinary tract infection (CAUTI) model has demonstrated that metabolic stimulation by substrates such as mannitol can enhance the efficacy of aminoglycosides in killing *E. coli*, including tolerant forms like biofilms [13, 14]. Moreover, the potentiation allows a reduction of aminoglycoside dosing [15]. Amikacin and mannitol are both approved for clinical use but exhibit differing safety profiles. Aminoglycosides such as amikacin can cause dose-dependent ototoxicity and nephrotoxicity [16]. In contrast, mannitol is used without major side effects and is not metabolized by host cells, being fully excreted by the kidneys. Pharmacological data suggest that low systemic doses of aminoglycosides achieve urine concentrations far exceeding the Clinical and Laboratory Standards Institute breakpoint for Enterobacteriaceae, and a single dose can sustain therapeutic levels against pathogens like *E. coli* for up to 3 days, making it a promising outpatient option [17, 18].

Based on the existing evidence, we designed a single-center randomized double-blinded pilot study (UROPOT: UROlogical infections and antibiotic POTentiation) with the aim of evaluating the clinical efficacy of metabolic potentiation in urinary tract antimicrobial therapy. Specifically, we will assess the combination of mannitol and low-dose amikacin for prophylaxis during endourological procedures in asymptomatic bacteriuria patients, comparing its prophylaxis efficacy to the current gold standard antibiotic prophylaxis regimen (ceftriaxone or amikacin standard dose alone). We will also assess its anti-biofilm activity compared to the gold standard regimens. UROPOT (ClinicalTrials.gov NCT05761405) is partially funded by the Leenaards Foundation’s Scientific prize and the University of Lausanne.

### Objectives

**Fig. 1:**
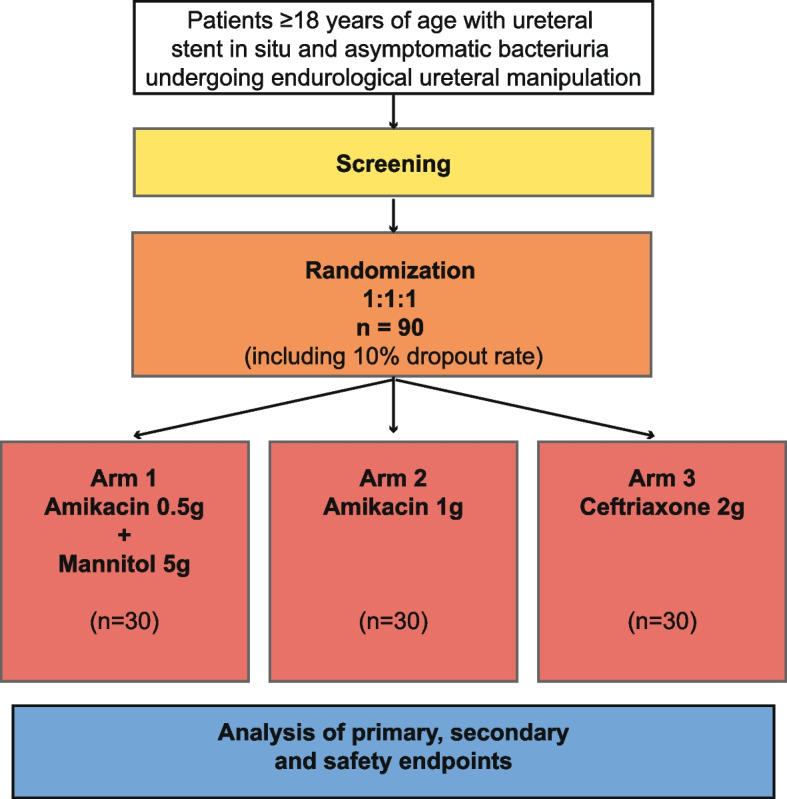
Screening process for patient eligibility for enrolment in the UROPOT study

UROPOT is a single-center randomized double-blinded pilot study. Patients will be allocated randomly to one of three treatment arms (with a 1:1:1 randomization ratio) to evaluate the efficacy of mannitol-potentiated reduced-dose amikacin in comparison to established gold standard treatments (ceftriaxone or amikacin standard dose alone; Fig. 1). The primary outcome of the study is to compare the incidence of postoperative infections assessed at 48 h postoperatively, defined by specific systemic symptoms and/or positive urine/blood culture. Secondary outcomes include assessing microbiological eradication through anti-biofilm activity, sustained microbiological eradication (urine cultures at 48 h and 14 days), and pharmacokinetics of mannitol and antibiotics in blood and urine samples. We will further evaluate the incidence of adverse events following amikacin/mannitol therapy and postoperative complications according to the Clavien-Dindo classification at postoperative day 14 (safety outcomes) [19].

### Trial design

UROPOT is a monocentric randomized double-blinded phase I/II exploratory trial that aims to assess mannitol-potentiated low-dose amikacin’s performance compared to gold standard prophylactic regimens in 90 patients over a 2-year enrolment period. Patients will be randomly assigned to one of three treatment arms (1:1:1 ratio; Fig. 1).

## Methods: participants, interventions, and outcomes

### Study setting

This single-center trial will take place at the Urology Department of the University Hospital of Lausanne, a tertiary university care center in Lausanne, Switzerland.

### Participants/eligibility criteria

Patients with asymptomatic *E. coli* and/or *K. pneumoniae* bacteriuria, undergoing endourological procedures. Inclusion and exclusion criteria determining the eligibility of study participants are reported in Table 1. Bacteriuria is defined as ≥ 10^2^ CFU/ml for both *E. coli* and *K. pneumoniae*. In case of mixed cultures, *E. coli* and/or *K. pneumoniae* should be significantly more enriched than other bacteria (i.e., ratios of quantitative results in CFU/ml of *E. coli*/other bacteria or *K. pneumoniae*/other bacteria are > 100).

**Table 1:** Inclusion and exclusion criteria of the UROPOT trial

Participants fulfilling all the following inclusion criteria are eligible for the study:
1. Written informed consent
2. Adults (≥18 years)
3. Patients with a ureteral stent in situ
4. Patients scheduled for endourological ureteral manipulations (e.g. endourological stone surgery, ureteral stent exchange)
5. Asymptomatic bacteriuria with strains of E. coli and/or K. pneumoniae sensitive to ceftriaxone and amikacin.
The presence of any one of the following exclusion criteria will lead to exclusion of the participant:
1. Allergy to one of the study drugs or excipients (Beta-lactams, aminoglycosides or mannitol)
2. Pregnant and lactating women
3. Glomerular filtration rate (CKD-EPI eGFR) < 50ml/min / 1,73m2
4. Middle to severe hearing impairment
5. Myasthenia gravis or other forms of myoneural disorders
6. Congestive heart failure, pulmonary oedema
7. Intracranial haemorrhage, compromised blood-brain barrier
8. Antibiotic treatment within 14 days prior to randomization
9. Mixed cultures of E. coli and/or K. pneumonia with other bacteria
10. Previous (within 3 months prior to randomization) or concomitant participation in another interventional clinical trial
11. Inability to understand and follow the protocol
12. Inability to give informed consent

### Who will take consent

Informed consent will be taken by the medical team (KS, FC, PB) or the study nurse as authorized surrogate (AM). Informed consent (supplementary data) will be sent by mail based on screening cultures and consent will be confirmed on visit 1 (see Table 2).

**Table 2:** Study visits

Time before/after day 0 (allocation)	− 30 days (± 15 days)	− 10 days (± 7 days)	− 5 days (± 4 days)	Day 0	Day 2 (± 24 h)	Day 14 (± 4 days)
**Visits**	**Pre-screening**	**Screening/information visit 0**	**Phone contact**	**Visit 1**	**Visit 2**	**Visit 3**
Assessment of potentially eligible patients	x					
**Assessment of eligibility during standard clinical consultation:**						
Inclusion/exclusion criteria check		x		x		
Participant information and delivery of informed consent		x				
Information about urinary culture results and feasibility to participate in the trial			x			
**Informed consent and randomization:**						
Signature informed consent				x		
Randomization allocation				x		
**Interventions:**						
Dispensation of study medication				x		
Transurethral intervention (ureteral stent manipulation)				x (+ 4–6 h)		
**Assessments:**						
Patient history, physical exam, pregnancy test		x				
Ureteral stent discomfort questionnaire		x			x	x
Vital signs (temperature, blood pressure, heart rate, and respiratory rate)		x		x	x	x
Blood draw for analyses: sodium, potassium, creatinine, eGFR (CKD-EPI), complete blood count		x^a^		x^b^	x	x^c^
Urine sampling for analyses: urine culture (microbiology)		x^a^		x^b^	x	x
**Assessment of anti-biofilm activity (secondary outcome)**				x		
Blood draw for analyses: aminoglycoside + mannitol level (AMK/Man level) at 1, 3, and 6 h after administration of drug				x		
Urine sampling for analyses: ceftriaxone, mannitol, and aminoglycoside levels in urine				x (during intervention)	x	
**Assessment of post-interventional clinical UTIs (primary outcome)**					x	X

^a^By phone

^b^Blood draw at 1/3/6 h post-infusion; urine culture intraoperatively

^c^Only if abnormal values at V2

### Study procedures

Patients with ureteral stents will undergo evaluation in the pre-surgical outpatient clinic, typically 2 weeks before endourological interventions, to assess laboratory and for urine culture. Following informed consent and a 24-h reflection period, patients qualifying for the trial based on urine culture results (see study population) will be randomly assigned to one of three antibiotic treatment arms (Fig. 1) at visit 1. The randomization list, maintained in the Pharmacy unit, will assign patient identification and randomization numbers to treatment infusion bags. The principal investigator will receive sealed envelopes for emergency unblinding to ensure confidentiality. Antibiotic prophylaxis, delivered as a single infusion over 30 min, will commence immediately upon receipt from the hospital pharmacy.

### Study intervention/allocation to blind treatment

The study will employ a pre-defined allocation table with balanced 1:1:1 to ensure equitable participant distribution, generated by the clinical research unit of the CHUV Pharmacy according to their standard process. The preparation of the study drug will be carried out by the unblinded pharmacist investigators. The intervention will consist in a single infusion over 30 min. The three treatment groups are:

1. Amikacin (low dose) + mannitol (arm 1): aminoglycosides (amikacin 500 mg, approximately 7.5 mg/kg; potentiated with mannitol 5 g). Thus, this combination therapy of aminoglycosides and mannitol reduces toxicity by decreasing the aminoglycoside dosing (potentiation of the activity of aminoglycosides by mannitol).
2. Amikacin (arm 2): a standard of antibiotic prophylaxis for endourological treatments (ureteroscopies, TURP, etc.). The dosage of 1000 mg is standard dosing for the average adult weight (ca. 15 mg/kg) [11, 22], a first-line treatment for urinary tract infections in Australia (*sahealth.sa.gov.au*).
3. Ceftriaxone (arm 3): a standard of care antibiotic prophylaxis in Switzerland. In clinical practice in Switzerland, asymptomatic bacteriuria is generally treated by antibiotics as recommended by IDSA guidelines [7]. The dosage of 2 g intravenously reflects the general practice.

### Criteria for discontinuation/modifying allocated intervention

As the intervention is a single infusion, discontinuation criteria do not apply.

### Relevant concomitant care permitted or prohibited

No antibiotics are allowed (exclusion criteria) immediately prior to or during the trial.

### Provisions for post-trial care

Additional lab parameters to monitor nephrotoxicity or tonometry for suspected ototoxicity will be made available to participants displaying adverse events in the context of the trial.

### Study endpoints

The primary outcome is the rate of postoperative infections 48 h postoperatively. Postoperative infections include systemic symptoms and positive urine/blood cultures matching preoperative urine cultures as deemed by the adjudication committee. Secondary outcomes include microbiological eradication via anti-biofilm activity assessed through catheter sonication, quantitative culture, and intraoperative urine culture; sustained microbiological eradication measured by urine cultures at postoperative days 2 and 14; and primary pharmacokinetic parameters of mannitol and antibiotics in blood and urine samples at specified time points. Safety outcomes will include monitoring the incidence of adverse events related to amikacin/mannitol combination therapy throughout the study, including serious adverse events (SAEs) and pre-specified adverse events of special interest such as ototoxicity and nephrotoxicity, along with vital signs. Additionally, postoperative complications at day 14 will be assessed using the Clavien-Dindo classification [19].

### Study procedures/participant timeline (Table 2)

During the screening visit (V0), patients with ureteral stents planned for endourological manipulation are preselected based on chart reviews every 2–4 weeks. A urine culture is taken as part of standard care. Written informed consent is obtained after a minimum 24-h reflection period, either on the day of intervention (for day-surgery) or the day before surgical intervention for pre-hospitalization, with results documented in both a screening log and the (eCRF). During visit 1 (V1), patients consenting to the study will undergo eligibility assessment by the investigator, followed by randomization conducted by pharmacy research staff. Data collected during the visit will be recorded in the eCRF, and patients will receive study treatment from the pharmacy as a single infusion bag, administered within 30 min of delivery, with intraoperative urine cultures and hardware extraction for culture, along with blood and urine samples for pharmacokinetic and antibiotic concentration quantitation. During visit 2 (V2), occurring 2 days post-intervention, patient history will be obtained regarding infectious complications; adverse events will be assessed and managed. Urine cultures and samples for antibiotic and mannitol analysis will be obtained, along with blood for safety laboratory assessments including electrolytes and creatinine, with all data recorded in the eCRF by research staff. During the end of study visit (V3), occurring 14 days post-intervention, patient history will be obtained regarding infectious complications, and adverse events will be assessed and managed. Urine cultures will be obtained, and blood may be drawn for safety laboratory assessments if deemed necessary based on pathological results from V2, with all data recorded in the eCRF by research staff.

### Sample size

As this is a phase I/II study, the sample size was chosen based on practical considerations and sufficient data to assess relevant safety issues (30 patients per group), accounting for potential drop-out rates considered within the feasibility assessment.

### Recruitment

Systematic screening of endourological procedures will be performed by the urology team (AM, KS, PB, FC). Patients will be contacted 1 month ahead of procedures to ensure culture sampling ca. 15 days ahead of time.

### Assignment of intervention

#### Blinding protocol

The study drugs will be prepared by the unblinded pharmacist investigators of the CHUV Pharmacy. The allocation sequence will be accessible through an interactive web response service (IWRS) embedded in the electronic case report form (eCRF9 (secuTrial®)), utilizing a pre-defined allocation table (block randomization) to ensure allocation concealment. Patients will be entered into the eCRF by the primary research team and transmitted to the pharmacy for randomization, with treatment prepared according to randomization and handed blindly to the research team. The randomization code will remain blinded to anyone involved in the study until the database is locked, and the allocation group will not be visible or accessible to assessors or patients throughout the study.

### Data collection and management

Data will be entered directly into the trial-specific electronic case report form (eCRF, secuTrial®), a standard electronic data capture system utilized by all clinical trial units (CTU) in Switzerland, with access granted after specific training. Biological sample results will be recorded in the participant’s hospital electronic file, and laboratory assessments will be conducted centrally at the Lausanne University Hospital (CHUV). Microbiological outcomes will be documented through sonication of extracted hardware [20] and intraoperative urine cultures, along with preoperative and postoperative urine cultures recorded for germ type and CFU count. Trial data will be directly entered into the trial-specific eCRF hosted at CHUV on secured servers, developed by Lausanne’s CTU under the SCTO-validated and Swissmedic-audited secuTrial® environment, with an audit trail system recording initial entries and changes. Authorized users can modify data in case of entry error, and data validation rules and automatic alerts will ensure data quality. The sponsor will implement and maintain quality assurance and control systems with written standard operating procedures (SOPs) to ensure compliance with the protocol, the International Conference on Harmonisation (ICH), Good Clinical Practice (GCP), and regulatory requirements, with system access granted after documented training and identification. Only authorized research staff, including the PI, will access and enter participants’ data in the eCRF using personal security passwords. Data alterations will be automatically traced in secuTrial® software, and the PI will validate the eCRF.

### Statistical analysis

The primary outcome is the proportion of postoperative infections within 48 h postoperatively. Fisher’s exact test will applied to compare the proportions between mannitol + low-dose amikacin arm vs full-dose amikacin arm and mannitol + low-dose amikacin arm vs ceftriaxone arm, respectively. To account for multiple comparisons, single-step method will be applied to control the family-wise error rate. Odds ratios with 95% confidence intervals and associated *p* value will be reported. Due to its exploratory nature and limited number of participants, no interim analysis will be performed. Microbiological eradication will be compared between the same groups mentioned in the primary analysis using *t*-tests. Difference in means with 95% confidence intervals and *p* values will be reported. If normality assumption is not met and the data approximately follow a log-normal distribution, data will be log-transformed before applying *t*-tests. In this case ratios of geometric means with 95% confidence intervals and *p* value will be reported. If none of the above methods hold, non-parametric methods will be considered.

Sustained microbiological eradication will be analyzed using mixed-effects models to account for the repeated measurements. Fixed effects will be the baseline microbiological eradication measured at D0, the intervention, the visit (D2 and D14), and the interaction between the intervention and the visit. Subject-specific random effect will be considered. Statistical assumptions will be assessed, and a similar methodology will be applied as for microbiological eradication to addressed them.

Primary pharmacokinetic parameters in blood (Cmax, AUC, t1/2) will be calculated for mannitol, amikacin, ceftriaxone, and for each intervention arm. In urine, descriptive statistics will be reported without any specific comparisons and spot urine concentrations.

Planned analyses will adhere to the sections detailing primary, secondary, and safety analyses, with a statistical analysis plan finalized and signed by all parties before code break. Datasets for analysis include the intention-to-treat (ITT), per-protocol (PP), and safety populations, with primary and secondary outcomes assessed in the ITT population and safety outcomes analyzed in the safety population.

For the primary outcome, we estimated an 8% premature discontinuation before 48 h post-surgery. Complete case analysis will be performed. Sensitivity analyses considering a best–worst-case scenarios will be applied. For the secondary outcomes, missing data mechanism will be considered at random and maximum likelihood-based methods will handle the missing data providing unbiased estimates.

### Oversight and monitoring

In this single-center trial, monitoring will be assumed by the clinical trial unit (EB) of the CHUV. Due to its exploratory nature and limited number of participants, no interim analysis will be performed.

Full review of key data (eligibility, primary outcome, and IMP administration) for 20% of the patient’s eCRF is planned in line with Swiss Clinical Trial Organization guidelines for risk-based monitoring and ICH-GCP. In view of the limited number of patients, of the well-established substances in the intervention and a nonetheless scheduled monitoring, a DMC was not deemed necessary.

Reporting of adverse events (AEs) or serious adverse events (SAEs) will be reported to the sponsor. SAEs will be reported within a 24-h period and any lethal SAE will be communicated within 7 days to the IRB. Evidence suggests that SAEs are not anticipated due to the long-standing use of medications. AEs such as transient nephrotoxicity or ototoxicity with aminoglycoside may be anticipated but are likely to be very infrequent due to the single use of this amikacin and its favorable profile compared to other aminoglycosides. Such AEs and SAEs will be reported to the Swiss Agency for Therapeutic Products (Swissmedic) as required indicating expectedness, serious-ness, severity, and causality.

### Patient and public involvement

The importance of the topic of antibiotic resistance and its incidence in patients undergoing repeated antibiotic prophylaxis as it is in long-term stent carriers was evaluated using questionnaires addressed to this target population. Their answers clearly showed that—in addition to the fear of antibiotic-associated side effects such as diarrhea—there is great respect for antibiotic resistance and multi-resistant germs. The question posed by the study is therefore also relevant to the general population.

### Dissemination plans

Data collected during the trial will be presented in conferences in concerned fields (infectious diseases, clinical pharmacology, and urology) and will be published in peer-reviewed journals.

## Discussion

Catheter-associated infections are a growing problem in healthcare. This is mainly due to the capacity of pathogens to form biofilms on foreign material [21]. Currently, the only antibiotics displaying activity against Gram-negative biofilms are quinolones [12]. However, a single mutation in the gyrase, the quinolones’ target, suffices to decrease a pathogen’s susceptibility [22]. Consistent with this, quinolones are ineffective in over 20% of cases due to rapidly increasing resistance, posing a significant challenge [23].

The lack of new antibiotics can be partially compensated by improving existing ones. Aminoglycosides are antibiotics with excellent activity and low bacterial resistance that are hampered by dose-dependent toxic effects in patients (nephrotoxicity and ototoxicity). They can be activated against prevalent Gram-negative pathogens (*E. coli*, *K. pneumoniae*) by specific metabolites, of which mannitol is highly interesting as it has not only a great impact on biofilms but is also nephroprotective. UROPOT is a first-in-class randomized trial of metabolism-based antimicrobial potentiation. The trial will test whether boosting the activity of aminoglycosides thereby enables the effective use of less toxic drug concentrations in patients with bacterial colonization and ureteral stent manipulation. Both amikacin and mannitol are readily available and cheap. Patient safety is high as all drugs used are well established. The potential side effects are well known and manageable and patient in whom potential harm could occur are excluded. The potential clinical impact, however, is significant, as new strategies for the rise in antibiotic resistance are urgently needed. Moreover, if anti-biofilm activity is confirmed, the application of the strategy beyond prophylaxis could be considered with clinical trials for complicated stent-associated urinary tract infections or infect-associated urolithiasis as a next step.

The potentiation of existing antibiotics represents a new and promising approach to circumventing bottlenecks of antibiotics development [14]. In addition, the combination of aminoglycosides with mannitol, as used here in the UROPOT trial, has the great advantage that the oto- and nephroprotective effect of mannitol can be utilized. On the one hand, the low dosage reduces potential side effects and toxicity of aminoglycosides, and on the other hand, mannitol seems to provide additional direct protection [15]. In addition to the effect of biofilm eradication, as demonstrated in preclinical models, the combination thus represents a unique protective and effective drug interaction which is the backbone of the UROPOT trial. UROPOT is already running at the Urology Outpatient clinic of CHUV.

A potential challenge in the UROPOT trial is achieving effective concentrations of amikacin and mannitol in the urinary tract for a sufficient period. Our current schedule may not allow a sufficient exposure for maximal effect to take place. To address this issue, bacterial strains derived from patients will be characterized in biomimetics for kinetics of potentiation within our consortium (JMK). This should identify potential strains refractory to potentiation and better calibrate dosing of antibiotics and metabolites in the urinary tract.

Logistically, the UROPOT trial does not face significant challenges as a single intervention is performed with 14-day follow-up consistent with standard of care. Adherence to treatment protocols is therefore simple and additional visits are straightforward. There is limited data collection and analysis, which should also make addressing potential safety concerns associated with the investigational therapy straightforward.

## Conclusion

The UROPOT trial presents a very promising approach poised to reshape clinical practices regarding antimicrobial prophylaxis in urological procedures. The combination of mannitol and low-dose amikacin holds potential as a future standard in antimicrobial prophylaxis for endourological interventions but potentially also for applications beyond prophylaxis.

## Trial status

The current version of the protocol version 2.0, 12.12.2023. The trial started on 15.02.2024, and the expected date for recruitment will be completed is 14.02.2026. Any modification of the protocol will be submitted for approval to the local IRB (CER-VD) and the Swiss Agency for Therapeutic Products (Swissmedic).

## Supplementary Information

Supplementary Material 1. SPIRIT checklist for Trials.

## Data Availability

After eCRF validation by the PI, the database will be locked, saved, and extracted for analysis. Data will be extracted into several clinical trial datasets (all together forming the clinical trial database) upon format requested by the statistician. The clinical trial datasets will be stored for 10 years on electronic folders secured on the CHUV servers and protected by passwords. The database will be archived for 10 years after study end.

## Abbreviations

eCRF: Electronic case report form
ITT: Intention-to-treat
